# The Heart and Circulation in Pregnancy

**Published:** 1905-04-08

**Authors:** 


### Hospital Clinics.

### THE HEART AND CIRCULATION IN PREGNANCY.

Upon this subject very conflicting observations and opinions have been recorded. Dr. Oliphant Nicholson[1] has subjected the whole question to a critical review, in which the doctrines of numerous observers are summarised and analysed. The starting-point of the discussion is the statement of Larcher, published in 1845, that in pregnancy the left ventricle undergoes hypertrophy, whilst the right ventricle and the auricles remain without change. This statement was based upon a series of post-mortem examinations made upon puerperal women at the Paris Maternity Hospital, but it is not supported by exact measurements, and generally it must be remembered that when death occurs during pregnancy or shortly after delivery there are usually complicating circumstances which may affect the size of the heart and may thus confuse the issue.

In opposition to Larcher's teaching may be quoted a recent series of clinical observations by Stengel and Stanton, who conclude that in pregnancy there is no increased work thrown on the left ventricle and no hypertrophy of its wall; the extension of the cardiac dulness to the left they interpret as a displacement of the organ upwards and to the left due to elevation of the diaphragm by pressure from below. On the other hand, these observers recognise a moderate dilatation of the right ventricle in the later months of pregnancy and due in all probability to the upward displacement of the diaphragm and to pressure on the lungs increasing the difficulties of the pulmonary circulation. They also recognise pulsation and sometimes a systolic murmur in the second and third left interspaces, and this they regard as an indication of a dilatation of the conus arteriosus and root of the pulmonary artery. Nicholson himself is satisfied that there is in pregnancy some hypertrophy of the left ventricle, but the degree of this varies in different cases.

Pregnancy without question throws considerable strain on the kidneys, and in view of the known relation between imperfect renal elimination and cardiac hypertrophy, such defect, even stopping short of actual disease, may well be in some measure responsible for an enlargement of the left ventricle. This argument adapts itself to the contention that in pregnancy there is retained in the body some agent which causes a widespread constriction of the arterioles. If it may be supposed that along with this constriction of the systemic arterioles there is (perhaps as a consequence) pulmonary engorgement and consequent strain upon the right ventricle there appears to be a fair explanation of what is often seen in pregnancy, namely, a slow and full radial pulse with a large pulsation in the jugular veins—the latter indicating a dilated right heart and the former suggesting a hypertrophied left ventricle. Such changes in the vascular circulation are very similar to those produced by suprarenal extract, and it may perhaps be suggested that this is produced in excess in pregnancy or that its normal action on the blood-vessels is not controlled in consequence of a defect in the thyroid secretion. According to Dr. Jas. Mackenzie a number of circulatory changes may be recognised as incidental to the pregnant state. There is, first, a "limitation of the field of cardiac response"; the inefficiency of the heart is shown by more or less inability to accommodate itself to the necessity for increased effort, and hence such symptoms as dyspnœa and palpitation on exertion are readily provoked. This may be noted at an early stage of pregnancy and long before the uterine contents can make an appreciable claim on the circulatory mechanism. The rate of the heart in early pregnancy is frequently increased, and change of position produces a more marked change in the rate than occurs in the non-pregnant state. In some cases a special form of pulse irregularity occurs due to premature contraction of the ventricle. As regards the size of the heart there is extension of the dulness to the right and sometimes also pulsation in this situation. Hence it must be concluded that there is enlargement of the right heart as well as some upward and outward displacement of the entire organ due to pressure on the diaphragm from below. With this engorgement of the right heart goes the tendency to pulmonary œdema, a greater or less degree of which can generally be found in the later months of pregnancy.

Another fact to which Dr. Mackenzie draws special attention is marked pulsation in the veins of the neck, which he regards as a further result of dilatation of the right heart. The tracing obtained from these pulsating veins is of great clinical importance, as it gives the key to the exact condition of the right heart, and suggests the appropriate management needed in cases of pregnancy complicated by valvular disease of the heart. An attempt to take a tracing from the jugular vein can hardly fail to be complicated by the impact of the carotid beat which will synchronise with the radial pulse, the sphygmogram of which should be recorded on the same smoked paper. Should the two tracings—that is, the radial pulse and the tracing obtained from the root of the neck — exactly coincide the beat from the root of the neck will be known to be due entirely to the carotid artery. But with a dilated right auricle there will be some regurgitation into the veins, and in this case the tracing over the root of the neck will show a small wave which will precede that due to the carotid beat. As further dilatation occurs this "auricular wave" becomes more and more prominent and may thus obscure the "carotid wave," which, however, can still be recognised by its coincidence with the radial pulse. In a yet more advanced stage a later wave may be detected, due to the contraction of the right ventricle. This does not occur, as at first might be thought possible, at the same moment as the carotid beat, though both are due to ventricular contraction. The ventricular venous wave is delayed because, before the stroke of the right ventricle can reach the jugular vein, it has to fill the right auricle, and only after this has been accomplished will it produce a pulse in the vein. In a yet further stage the auricle may be so distended that it has only slight power of contraction, and in these circumstances there will be a small auriclar venous wave whilst the tricuspid regurgitation with an easily filled auricle will mean a considerable and early ventricular wave. And as a final stage there may be complete paralysis of the right auricle with loss of the auricular wave from the venous tracing and a single large wave due to and synchronous with the contraction of the right ventricle. These more extreme forms of venous pulse only occur in cases of pregnancy complicated with mitral disease. In the last form it is evident that the right auricle is incapable of contracting. With this it is in the highest degree probable that there is also no contraction of the left auricle, and hence with a ventricular venous pulse there will be disappearance in a case of mitral stenosis of the pre-systolic murmur, whilst a long diastolic murmur will mean the rush of blood through a narrowed mitral orifice during the ventricular diastole. These observations are of great value in relation to the question of pregnancy in women known to be the subjects of mitral disease. Mackenzie advises that with manifest failure of compensation (dropsies, enlarged liver, etc.) pregnancy should be forbidden. He also applies the same prohibition to cases with fair compensation where there is paralysis of the auricle as shown by the presence of a diastolic murmur and the absence of a pre-systolic murmur, or of a continued irregularity of the pulse, or of a jugular pulse of the ventricular type. With fair compensation, the apex beat within the nipple line and due to the left ventricle, and a pre-systolic or systolic mitral murmur, pregnancy may be permitted. A clinical contribution to this question was made some years ago by Dr. Geo. Middleton [2] and may be consulted with advantage by those interested in the subject. In this paper statistics are set forth to controvert the gloomy views advanced by the late Dr. Angus Macdonald in his book on "Heart Disease during Pregnancy."

[1] Jour. of Obs. and Gyn., Jan., 1905. [2] Lancet, Oct. 26 and Nov. 2, 1889.

